# Serum Free Light Chain Only Myeloma with Cytoplasmic IgM

**DOI:** 10.1155/2014/676913

**Published:** 2014-06-17

**Authors:** Hideaki Ebana, Ken-ichi Nakamura, Yoshihiro Nozawa, Ritsuko Seki, Masayuki Mita

**Affiliations:** ^1^Division of Hematology/Oncology, Shirakawa Kosei General Hospital, 2-1 Toyochi Kamiyajirou, Shirakawa, Fukushima 961-0005, Japan; ^2^Division of Diagnostic Pathology, Shirakawa Kosei General Hospital, 2-1 Toyochi Kamiyajirou, Shirakawa, Fukushima 961-0005, Japan; ^3^Medicine/Hematology and Oncology, Kurume University School of Medicine, 67 Asahimachi, Kurume, Fukuoka 830-0011, Japan

## Abstract

In the past decade, the serum free light chain (FLC) immunoassays have become widely available enabling greater sensitivity in the diagnosis and management of monoclonal light chain diseases. Here, we describe a rare case of serum free light chain only myeloma with cytoplasmic IgM. A 75-year-old woman presented with a progressively worsening lumbosacral pain. FDG PET/CT images showed increased FDG uptake in the sacral mass, vertebral bodies, and ribs. Laboratory data found hypogammaglobulinemia and the bone marrow aspirate revealed only 2.2% of plasma cells. The serum and urine protein electrophoresis did not detect a monoclonal band. However, the serum FLC immunoassays reported an abnormal kappa/lambda ratio (0.001) indicating the presence of monoclonal lambda FLC. The sacral tumor biopsy revealed proliferation of plasma cells and immunohistochemical staining showed that the plasma cells were positive for CD138, IgM, and lambda light chain but negative for CD20. This case may have previously been described as a nonsecretory IgM myeloma but recently would be identified as free light chain only myeloma. The immunohistochemical and genetic features of the clonal plasma cells in free light chain only myeloma need to be further investigated to better understand the relevance and incidence of this myeloma type.

## 1. Introduction

During the past decade the serum free light chain (FLC) immunoassays have become widely available enabling greater sensitivity in the diagnosis and management of monoclonal light chain diseases [1, 2]. These assays have proved to be particularly beneficial for the detection of monoclonal FLCs in patients previously considered to be nonsecretory according to electrophoresis [3]. Such patients can now be reclassified as measurable serum free light chain only myeloma [4, 5]. A recent study by the Asian Myeloma Network reported that light chain myeloma and nonsecretory myeloma account for approximately 18% and less than 2% of all myelomas, respectively [6]. Furthermore, the diagnostic performance of the serum FLC immunoassays and immunohistochemistry has been evaluated in patients with IgM paraproteinemia, particularly Waldenström's macroglobulinemia (WM) and IgM monoclonal gammopathy of undetermined significance (MGUS). There have been very few cases of myeloma with cytoplasmic IgM documented [7–9]. Here, we present a very rare case of serum free light chain only myeloma with cytoplasmic IgM. This diagnosis was confirmed by immunohistochemistry, serum FLC immunoassays, magnetic resonance imaging (MRI), and ^18^F-fluorodeoxyglucose (FDG) positron emission tomography/computed tomography (PET/CT) imaging.

## 2. Case Presentation

**Figure 1:**
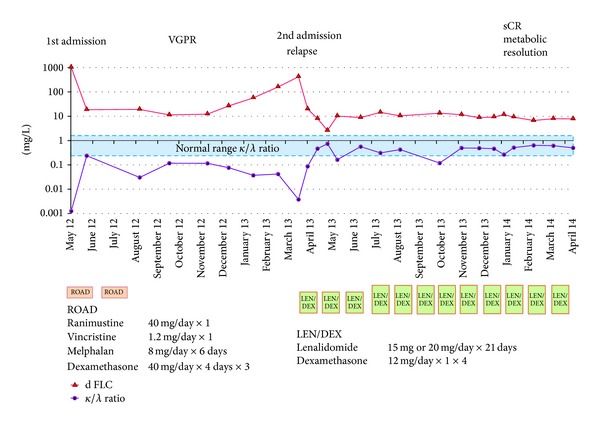
Clinical course of the patient and response to treatment. Difference between involved and uninvolved free light chain (dFLC) and *κ*/*λ* ratio are reported in the graph alongside the clinical course and treatment regimens.

**Figure 2:**
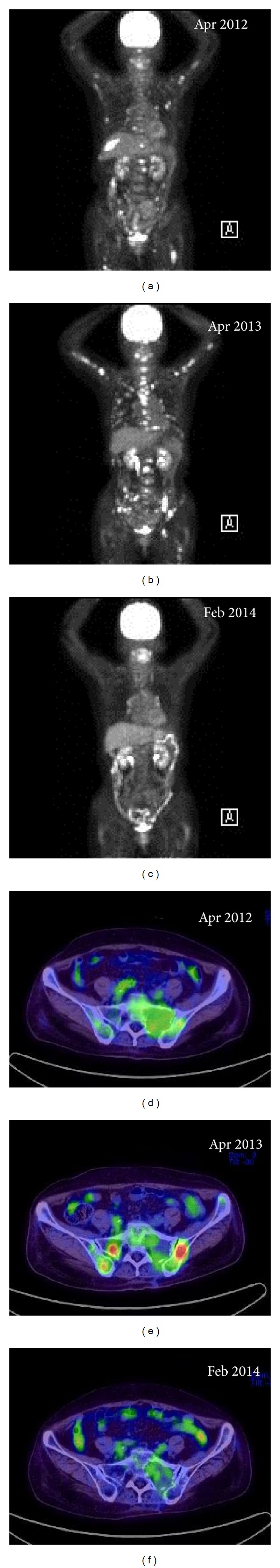
Coronal and axial fused ^18^F-FDG PET/CT images. (a) Initial diagnosis of myeloma, multiple foci of metabolic activity. (b) Extension of lesions and new lesions following relapse. (c) Complete metabolic resolution of myeloma. (d) In initial diagnosis of myeloma, PET/CT image shows ^18^F-FDG avid lytic lesions sacrum and iliac bone involvement. (e) In relapsed myeloma, PET/CT image shows developed ^18^F-FDG avid iliac and sacral bone lesions. (f) After 10 months undergoing LEN/DEX therapy, PET/CT image shows complete resolution of the metabolic activity.

**Figure 3:**
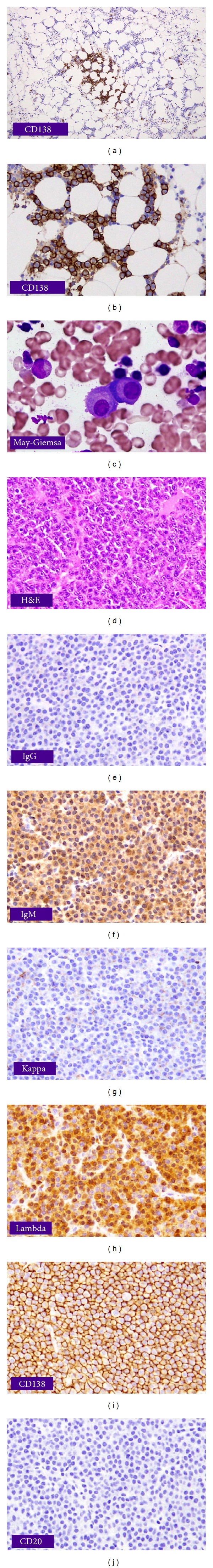
Bone marrow and specimen from the sacral mass. ((a), (b)) Immunostaining for CD138 in the bone marrow clot section showing microcluster infiltration of plasma cells (x40, x200). (c) Bone marrow aspiration smear showing atypical plasma cells (May-Giemsa stain x600). ((d)–(j)) Specimen of sacral mass. (d) Hematoxylin and eosin (H&E) stain showing diffuse infiltration of plasma cells. Immunohistochemical stain showing (f) IgM+, (h) lambda light chain+, (i) CD138+, (e) IgG−, (g) kappa light chain−, and (j) CD20−.

A 75-year-old woman presented with a 6-month history of progressively worsening lumbosacral pain. She was suspected of having myeloma or metastatic bone disease, based on both CT and MRI scans. The CT scan images revealed osteolytic lesions in the ribs and the sacrum without hepatosplenomegaly and lymphadenopathy. The MRI of the lumbosacral spine revealed a heterogeneously enhancing, destructive sacral mass, with focal and diffuse infiltration of the lumbar vertebrae. The laboratory blood values were as follows: hemoglobin of 9.5 g/dL, red blood cells of 2.62 × 10^12^/L, white blood cells of 3.4 × 10^9^/L, platelets of 150 × 10^9^/L, total protein of 5.23 g/dL (normal range, 6.30–8.20), albumin of 3.39 g/dL (normal range, 4.20–5.40), and elevated *β*2-microglobulin of 4.5 mg/L (normal range, 0.8–2.4). There was no evidence of a monoclonal protein (M-protein) detected by either serum or urine immunoelectrophoresis and the immunoglobulin concentrations were low, indicating hypogammaglobulinemia: IgG: 435 mg/dL (normal range, 870–1700), IgA: 16 mg/dL (normal range, 110–410), IgM: 11 mg/dL (normal range, 34–250), and IgD: 0.6 mg/dL (normal range, 0–9). In view of the diagnostic difficulties, serum FLC measurements were requested, with the following results: *κ* FLC concentration of 1.3 mg/L (normal range, 3.3–19.4); *λ* FLC concentration of 1030.0 mg/L (normal range, 5.7–26.3); and serum *κ*/*λ* ratio of 0.001 (normal reference range, 0.26–1.65) (Figure 1). These results indicated the presence of monoclonal *λ* FLCs, and a diagnosis of myeloma was considered. However, the bone marrow aspirate revealed infiltration with only 2.2% of plasma cells and the chromosomal analysis demonstrated a normal 46,XX karyotype. ^18^F-FDG PET/CT was performed in order to clarify the diagnosis. It highlighted multiple areas of anomalous concentration of ^18^F-FDG in the sacrum, iliac bone, pubic, ribs, bilateral femora, and the vertebrae at C2, Th9, Th10, L1, and L2, with a standard uptake value (SUV) between 3.7 and 17.6 (Figures 2(a) and 2(d)). These findings were considered indicative of neoplastic disease and a tissue biopsy was recommended. The sacral tumor open biopsy was performed by an orthopedic surgeon. Hematoxylin and eosin-stained sections of the biopsy specimen revealed marked proliferation of plasma cells and immunohistochemical staining showed that the plasma cells were positive for CD138, IgM, and *λ* light chain but negative for CD20, CD3, IgG, IgA, IgD, and *κ* light chain (Figures 3(d)–3(j)). Due to the identification of cytoplasmic IgM, genetic analysis was performed on paraffin-embedded biopsy specimens to rule out WM and IgM MGUS. The MYD88 L265P mutation has been reported to be expressed in both WM and IgM MGUS patients but not myeloma [10]. In the biopsy specimen, MYD88 L265P mutation was not detected by real-time allele-specific polymerase chain reaction (AS-PCR) assays. These results supported the fact that this case was neither WM nor IgM MGUS. Therefore, a diagnosis of MM was confirmed (Durie-Salmon stage IIIA, International Staging System II). The patient was treated initially with ROAD (ranimustine, vincristine, melphalan, and dexamethasone) regimen as the alkylating agent ranimustine (approved for use in Japan) has been reported to have tolerable side effects [11]. Following 2 cycles of ROAD therapy, her general condition and lumbosacral pain improved. Her disease progression was monitored serially using the serum FLC immunoassays, which showed a reduction in the *λ* FLC concentration from 1030 mg/L at diagnosis (May 2012) to 19.7 mg/L (August 2012). She achieved a very good partial remission (VGPR), defined as a >90% decrease in the dFLC (difference between involved and uninvolved serum FLC levels) [12]. In April 2013, radiographs revealed an undisplaced pathological fracture of the right upper arm bone. A second bone marrow aspirate was also normal with 3.2% infiltration of plasma cells; however the bone marrow clot section identified a microcluster infiltration of plasma cells (Figures 3(a)–3(c)). At this time, the serum *λ* FLC levels had increased to 422.0 mg/L and the *κ*/*λ* ratio was abnormal (0.004) indicating the return of the monoclonal disease. In agreement with these results, a coronal PET/CT scan highlighted multiple foci of metabolic activity and confirmed the clinical relapse (Figures 1, 2(b), and 2(e)). The patient was treated with a regimen of lenalidomide/dexamethasone (LEN/DEX) every 4 weeks. In July 2013, the serum *λ* FLC concentration normalized (20.1 mg/L) alongside the *κ*/*λ* ratio (0.562). In February 2014, 10 months following the initiation of LEN/DEX therapy, the PET/CT images showed complete resolution of the metabolic activity, indicating successful treatment, with the SUV max between 1.8 and 2.3 within the stable lytic bone lesions. At this time, the patient achieved a stringent complete response (sCR) by serum FLC immunoassay, correlating with the PET/CT images (Figures 1, 2(c), and 2(f)).

## 3. Discussion

It is noteworthy that there were two significant findings in our case. Firstly, the sacral tumor biopsy revealed marked proliferation of plasma cells and the immunohistochemical staining of the clonal plasma cells were positive for both *λ* light chain and IgM. Secondly, the bone marrow aspirate was unable to detect the clonal plasma cells even though the CT, MRI, and PET/CT showed the presence of multiple anomalous bone lesions.

In this case, although an M-protein could not be detected by serum or urine electrophoresis, the serum FLC assay detected elevated *λ* FLCs and an abnormal *κ*/*λ* FLC ratio indicating the presence of monoclonal *λ* FLCs. Significantly, immunohistochemical studies on the sacral tumor biopsy demonstrated the presence of cytoplasmic IgM in the plasma cells, indicating immunoglobulin synthesis. Therefore, the clonal plasma cells produced both FLC and IgM but only the FLCs were detected in the serum. Although our case may have previously been described as a nonsecretory IgM myeloma, the diagnosis of “measurable serum free light chain only myeloma with cytoplasmic IgM” is more appropriate [4, 5]. There have been very few cases of plasma cell neoplasms with cytoplasmic IgM described. However, one such example was a case of nonsecretory plasma cell leukemia/myeloma that expressed IgM heavy chain but was negative for *κ* and *λ* light chains by immunostaining, with no detectable serum IgM paraprotein [7–9]. IgM myelomas are rare and account for 0.3% of all myelomas and 1.2% of patients with all IgM paraproteinemia, which include WM, B-cell lymphoma, MGUS, IgM myeloma, *μ*-heavy chain disease, and lymphoproliferative disorders [13, 14]. In such cases, immunohistochemistry can be an important tool to help distinguish myeloma from these other neoplasms, in particular WM [15]. In our case, CD20, which is a characteristic immunophenotype for WM, was negative. It has been noted that typical clinical features, such as lytic bone lesions in myeloma or organomegaly in WM, are not always present to help with the distinction and therefore additional diagnostic tools are necessary for a definitive diagnosis of myeloma [16]. MYD88 L265P mutation is a potential new biomarker with diagnostic, prognostic, and possible therapeutic implications. By means of AS-PCR, the MYD88 L265P mutation is detectable in 93% of patients with WM and in 54% with IgM-MGUS but is absent in myeloma samples [10]. In our case, MYD88 L265P mutation was not detected. Taken together, these results were consistent with a diagnosis of MM.

The second significant finding was that although multiple bone lesions were detected by PET/CT and the sacral tumor biopsy revealed proliferation of plasma cells, repeat bone marrow aspirates showed only 2-3% of plasma cells. There could be a number of reasons for this discrepancy including the effect of blood dilution, marrow fibrosis, or sample clotting [17]. However, in our case the normal aspirates were most likely due to focal disease distribution. This was illustrated by the CD138 immunohistochemical staining of the bone marrow clot section. This highlighted microclusters of plasma cells, more resembling an aspirate from an MGUS patient [17]. Furthermore, the MRI scans identified neoplastic lesions that were very heterogeneous and were composed of small clusters, large clusters, and expansile masses of plasma cells. This case highlights the fact that a bone marrow aspirate alone may be insufficient to confirm a diagnosis of myeloma and a biopsy should be considered in plasma cell disorders.

## 4. Conclusion

Our case was diagnosed as “serum free light chain only myeloma with cytoplasmic IgM,” and confirmed the existence of a rare myeloma type. The immunohistochemical and genetic features of the clonal plasma cells in free light only myeloma need to be further investigated to better understand the relevance and incidence of this myeloma type.
